# Associations of 2923 plasma proteins with incident atopic dermatitis in a prospective cohort study and genetic analysis

**DOI:** 10.1097/MD.0000000000043447

**Published:** 2025-07-18

**Authors:** Sui Deng, Miaoyi Zhang, Tongtong Zhang, Rui Mao

**Affiliations:** a Changde Hospital, Xiangya School of Medicine, Central South University (The First People’s Hospital of Changde City), Changde, China; b Clinical Medicine, School of Clinical Medicine, Dali University, Dali, China; c Obesity and Metabolism Medicine-Engineering Integration Laboratory, Department of General Surgery, The Third People’s Hospital of Chengdu, Affiliated Hospital of Southwest Jiaotong University, Chengdu, China; d Department of Dermatology, Xiangya Hospital, Central South University, Changsha, China.

**Keywords:** atopic dermatitis, biomarker, genetics, Mendelian randomization, plasma proteomics, prospective studies

## Abstract

The relationship between proteomics and atopic dermatitis (AD) remains underexplored but holds significant potential for therapeutic intervention. We analyzed data from a longitudinal cohort of 51,458 UK Biobank participants to investigate the relationship between AD risk and serum levels of 2923 proteins. Multivariate Cox regression was initially applied to evaluate associations between protein concentrations and AD incidence. Subsequently, two-sample Mendelian randomization (MR), summary-data-based MR, and colocalization analyses were conducted to establish genetic associations with AD. Our analysis identified 23 proteins significantly associated with AD risk. Two-sample MR further validated ten proteins exhibiting robust causal relationships with AD. Comprehensive assessments using summary-data-based MR, colocalization, and differential expression analyses pinpointed 5 key proteins – CACYBP, CETN3, MOCS2, TNFAIP8, and PVALB – with potential protective effects against AD. A novel protein-based scoring system, integrating these biomarkers with inflammatory markers, achieved superior predictive accuracy for AD onset (area under the curve = 0.833), outperforming both the polygenic risk score and eosinophil percentage. This extensive proteomic and genetic study within a European adult cohort provides compelling causal evidence for several proteins as potential biomarkers for AD, offering new avenues for early diagnosis and therapeutic development.

## 1. Introduction

Atopic dermatitis (AD) continues to impact a substantial segment of the global population.^[1]^ The pathogenic mechanisms of AD are complex and not fully understood. Currently, management strategies primarily involve immunosuppressants and broad-spectrum anti-inflammatory drugs. While these treatments alleviate symptoms, they often fail to prevent recurrence, leading to significant patient distress and considerable healthcare expenditures. Furthermore, therapies targeting Th2-type and non-Th2-type inflammatory pathways in AD are limited by modest efficacy and increased risks of infections and malignancies.^[2,3]^ This underscores the critical need for the identification and development of novel therapeutic targets.

In our current investigation, we examined extensive proteomic data collected from 51,458 individuals participating in the UK Biobank’s Plasma Protein Profiling Initiative (UKB-PPP). Our primary objective was to conduct an in-depth assessment of potential correlations between 2923 distinct plasma proteins and the predisposition to develop AD. Through the integration of these protein indicators with established inflammatory markers, we successfully created 2 novel assessment tools: a protein-derived scoring mechanism and an advanced risk assessment framework. These innovations have substantially enhanced our capability to predict the likelihood of AD development with greater precision.

## 2. Methods

### 2.1. Study design and participants

**Figure 1.**
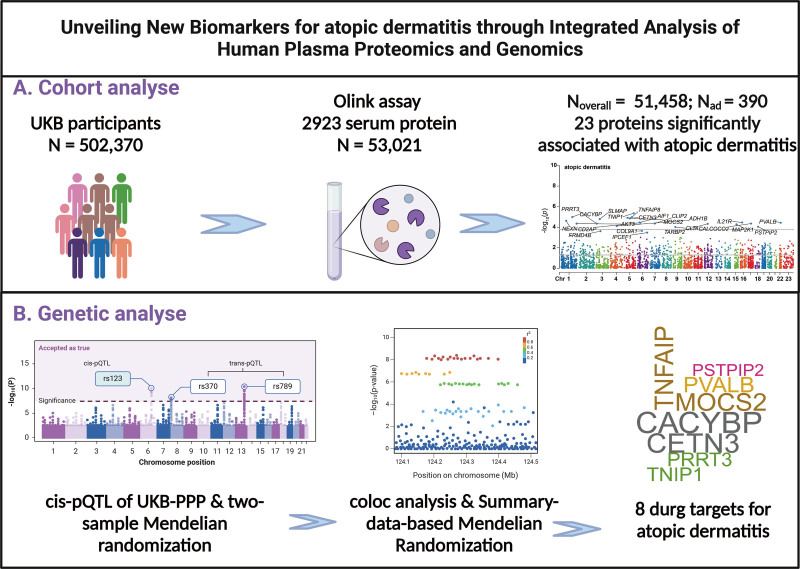
Research design and flowchart.

The study’s methodology is depicted in Figure 1. Drawing upon the UK Biobank initiative – a longitudinal population study encompassing around 502,370 individuals aged 40 to 69 years – data were collected from 22 regional centers throughout the United Kingdom during the period of 2006 to 2010.^[4]^ Initial enrollment involved obtaining written consent, conducting thorough clinical assessments, and gathering extensive information regarding participants’ social demographics, health behaviors, clinical background, pharmaceutical usage, and biological specimens. Longitudinal health monitoring was implemented through national healthcare databases, with follow-up continuing until July 2023.

The proteomic analysis component, known as the UK Biobank Plasma Protein Profiling Initiative (UKB-PPP), received collaborative funding from 13 biotechnology and pharmaceutical organizations.^[4,5]^ From the initial pool, 54,306 subjects were selected for proteomic profiling. After implementing stringent quality assessment protocols, proteomic data from 51,458 participants met the established criteria for inclusion in subsequent analyses. The study protocol received ethical clearance from the North West Multi-center Research Ethics Committee (Reference: 21/NW/0157), with all research activities and data processing performed in compliance with the operational framework specified in UK Biobank Application 93810.

### 2.2. Protein quantification and the processing of proteomic data within the UK Biobank

The technical specifications and analytical approaches for proteomic assessment and subsequent data handling within the UKB-PPP have been thoroughly documented in previous publications.^[6]^ During quality control procedures, 3 protein markers – GLIPR1 (99.7% missing values), NPM1 (74.0% missing values), and PCOLCE (63.6% missing values) – were removed from further consideration due to excessive data incompleteness. For the retained 2920 proteins, missing value estimation was performed using the k-nearest neighbors algorithm (k = 10) as implemented in previous studies.^[7]^ Subsequently, all protein measurements underwent Z-score transformation to normalize the data distribution and facilitate comparative analyses across different protein targets.

### 2.3. Outcome assessment

The determination of clinical outcomes in our investigation primarily utilized national healthcare databases, incorporating inpatient records from 3 major sources: the English Hospital Episode Statistics, Welsh Patient Episode Database, and Scottish Morbidity Records. Case identification for AD followed the standardized diagnostic criteria specified in the ICD-10 classification system (Code L20). To maintain cohort integrity, we excluded individuals with either patient-reported AD diagnoses or hospital-based AD confirmations recorded during the baseline assessment phase. The observation window for each study participant spanned from their initial enrollment date through July 1, 2023.

### 2.4. Proteomic association analyses

Our primary statistical approach employed Cox regression modeling to examine potential correlations between plasma protein concentrations and AD development. The analytical framework incorporated comprehensive adjustments for multiple demographic and clinical variables to minimize confounding influences. Socioeconomic factors were accounted for through multiple indicators: annual household income was stratified into 6 brackets ranging from under £18,000 to over £100,000, with an additional category for unreported income levels. Ethnic background was classified into 5 groups: Caucasian, South Asian, African-Caribbean, mixed ancestry, and other ethnicities. Educational background was categorized according to the highest qualification achieved, ranging from basic certificates to advanced degrees, with an option for no formal qualifications.

Lifestyle factors were incorporated through several parameters: tobacco use was classified as current smoker, former smoker, or never smoker; alcohol consumption frequency was divided into 4 levels from abstinent to daily intake. Physical activity levels were expressed in metabolic equivalent minutes per week (MET-min/week) as a continuous variable. To account for potential seasonal variations in protein levels, the timing of blood sample collection was categorized into 4 meteorological seasons based on the UK climate pattern.

### 2.5. Mendelian randomization (MR) analysis

The investigation’s methodological framework rigorously followed the strengthening the reporting of observational studies in epidemiology (STROBE-MR) reporting standards for genetic epidemiological studies, with complete documentation available in eTable 1,^[8]^ Supplemental Digital Content, https://links.lww.com/MD/P444. Genetic instruments and protein quantitative trait loci (pQTL) data were exclusively obtained from publicly accessible genome-wide association study repositories that had received appropriate ethical approvals. Detailed metadata regarding these genetic resources are comprehensively presented in eTable 2, Supplemental Digital Content, https://links.lww.com/MD/P444. Within our analytical framework, circulating protein concentrations measured through the UK Biobank Plasma Profiling initiative were designated as the exposure metric, while AD diagnosis served as the primary clinical endpoint. Genetic causal inference analyses were performed using the TwoSampleMR computational package, implementing distinct analytical approaches based on the number of available genetic instruments. For proteins with single pQTL associations, we applied the Wald ratio estimator, whereas proteins with multiple independent genetic variants were analyzed using the inverse-variance weighted MR approach. Effect estimates were expressed as odds ratios corresponding to a 1 standard deviation (SD) increment in protein concentration.

To establish the reliability and generalizability of our findings, we performed extensive external validation using aggregated genetic association statistics from multiple independent populations. This validation framework incorporated data from 3 major sources: (1) the DECODE genetic study involving 35,559 Icelandic individuals,^[9]^ (2) a Finnish population cohort of 10,708 subjects analyzed by Pietzner and colleagues,^[10]^ and (3) a large-scale meta-analysis cohort compiled by Zheng et al,^[11]^ which synthesized findings from 5 previously published genome-wide association study investigations. The selection of genetic instruments and the exclusion of confounders (eFigure 1, Supplemental Digital Content, https://links.lww.com/MD/P444) are described in supplement 1 (emethods, Supplemental Digital Content, https://links.lww.com/MD/P444).

To further strengthen causal inference, we employed Steiger filtering to validate the temporal directionality of protein-outcome associations.^[12]^ Comprehensive methodological details for advanced analyses – including Bayesian co-localization, summary-data-based MR, Phenome-Wide MR, single-cell transcriptomic profiling (eTable 3, Supplemental Digital Content, https://links.lww.com/MD/P444), and polygenic risk score (PRS) derivation – are extensively documented in supplement 1 (emethods, Supplemental Digital Content, https://links.lww.com/MD/P444).^[4,13–21]^

### 2.6. Development of the protein scoring system

The study population was systematically partitioned into development and validation subsets through random allocation at a 7:3 ratio. Within the training cohort, individual proteomic risk scores were computed using a Cox proportional hazards framework, mathematically represented as: ${\hat{h}}_{i}\left(t\right)={\hat{h}}_{0}\left(t\right)exp\left(x_{i}^{\prime }\hat{\beta }\right)$, where exp denotes protein expression levels; β represents the multivariate-adjusted regression coefficients; and $h_{0}\left(t\right)$ signifies the baseline hazard function.^[22]^ Based on the median proteomic risk score, participants were categorized into elevated-risk and reduced-risk strata, enabling comparative analysis of AD incidence through cumulative hazard function evaluation. To optimize predictive accuracy, we implemented a sequential modeling strategy. The initial model incorporated an extensive panel of inflammatory indicators, including absolute neutrophil counts, eosinophil concentrations, neutrophil-to-lymphocyte ratio, platelet-to-lymphocyte ratio, and additional proteomic markers – totaling 11 candidate predictors. These variables underwent lasso regularization to enhance model specificity and prevent overfitting through automated feature selection. The 4 most informative predictors identified through this process were subsequently integrated into a multivariate Cox regression framework to generate refined risk estimates. Model performance was quantitatively assessed using time-dependent receiver operating characteristic analysis, implemented through the SurvivalROC package in R. The predictive capacity was quantified by calculating the area under the receiver operating characteristic curve (ROC) (area under the curve [AUC]) across various time points.

### 2.7. Statistical analysis

The investigation employed a multi-tiered statistical approach to examine proteomic–AD associations. Initial exploratory analyses incorporated the Benjamini–Hochberg correction method to account for multiple hypothesis testing, maintaining the false discovery rate (FDR) below 0.05 as the significance threshold. For causal inference analyses using MR, a conventional *P*-value cutoff of .05 was implemented to establish statistical significance. All computational procedures were executed using R statistical software (v4.3.1).

## 3. Results

Our study analyzed data from 51,458 participants enrolled in the UKB-PPP, predominantly of white ethnicity, comprising 47,548 participants (92%). The mean age of the cohort was 56.8 years, with a SD of 8.2 years. The follow-up period for the cohort had a median duration of 14.3 years, with an interquartile range of 13.6 to 15.0 years (Table 1).

**Table 1:** Baseline characterization of participants in UKB.

Variable	Overall, N = 51,458	No Atopic dermatitis, N = 51,068	Atopic dermatitis, N = 390	*P*-value*
Age, Mean (SD)	56.81 (8.22)	56.81 (8.21)	56.75 (8.43)	>.9
Sex, n (%)				.029
Female	27,661 (54%)	27,430 (54%)	231 (59%)	
Male	23,797 (46%)	23,638 (46%)	159 (41%)	
BMI, Mean (SD)	27.48 (4.81)	27.48 (4.80)	27.60 (5.05)	>.9
Race, n (%)				.3
White	47,548 (92%)	47,188 (92%)	360 (92%)	
Asian_or_Asian_British	1862 (3.6%)	1845 (3.6%)	17 (4.4%)	
Black_or_Black_British	312 (0.6%)	311 (0.6%)	1 (0.3%)	
Mixed	952 (1.9%)	948 (1.9%)	4 (1.0%)	
Chinese	144 (0.3%)	141 (0.3%)	3 (0.8%)	
Other ethnic group	640 (1.2%)	635 (1.2%)	5 (1.3%)	
smoking_status, n (%)				.2
Current	5477 (11%)	5425 (11%)	52 (13%)	
Previous	18,029 (35%)	17,891 (35%)	138 (35%)	
Never	27,952 (54%)	27,752 (54%)	200 (51%)	
Alcohol_intake_frequency, n (%)				.4
Never	4468 (8.7%)	4426 (8.7%)	42 (11%)	
Occasionally	11,733 (23%)	11,646 (23%)	87 (22%)	
Sometimes	24,896 (48%)	24,717 (48%)	179 (46%)	
Daily	10,361 (20%)	10,279 (20%)	82 (21%)	
Summed_MET, Mean (SD)	2651.46 (2654.42)	2652.50 (2655.61)	2514.92 (2493.17)	.3
Townsend, Mean (SD)	−1.17 (3.19)	−1.17 (3.19)	−1.54 (2.97)	.027
Income, n (%)				.9
Less_than_18,000	13,469 (26%)	13,366 (26%)	103 (26%)	
18,000_to_30,999	13,564 (26%)	13,458 (26%)	106 (27%)	
31,000_to_51,999	12,572 (24%)	12,486 (24%)	86 (22%)	
52,000_to_100,000	9349 (18%)	9274 (18%)	75 (19%)	
Greater_than_100,000	2504 (4.9%)	2484 (4.9%)	20 (5.1%)	

SD = standard deviation.

* Wilcoxon rank sum test; Pearson Chi-squared test.

### 3.1. Observational associations of proteins with AD

**Figure 2.**
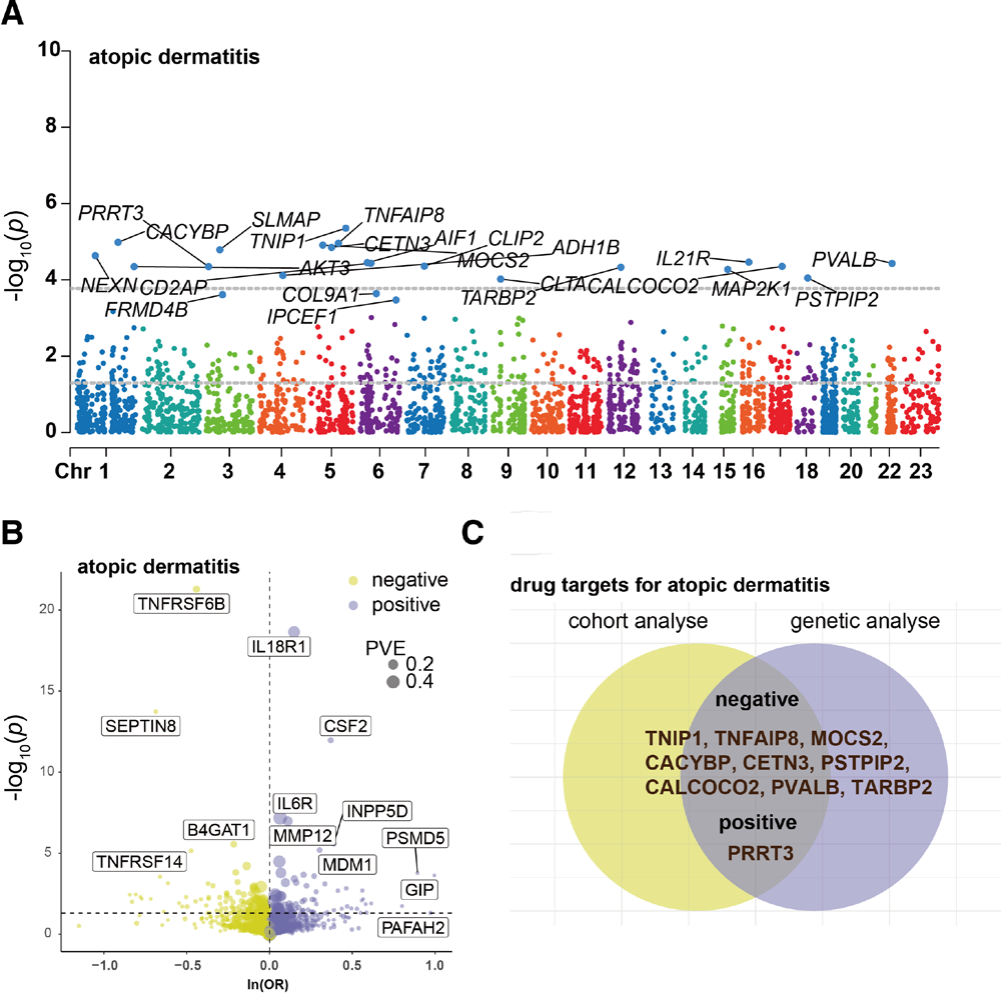
Discovery of potential biomarkers for atopic dermatitis through integrative cohort and Mendelian randomization analyses. (A) Genome-wide protein association landscape: the Manhattan plot displays protein-disease associations across the human genome, with each point representing a protein mapped to its corresponding gene locus (*x*-axis) and its association significance (−log10 transformed *P*-values on *y*-axis). The horizontal dashed lines demarcate the significance threshold (*P* < .05), with proteins surpassing FDR correction highlighted in blue and annotated with their respective names. (B) Causal effect estimation: the volcano plot illustrates results from cis-oriented Mendelian Randomization analysis, plotting effect estimates (*x*-axis) against their statistical significance (−log10 *P*-values on *y*-axis). Proteins demonstrating significant causal relationships with atopic dermatitis are positioned beyond the vertical significance threshold, with effect directionality indicated by their horizontal placement. (C) Integrative biomarker identification: a Venn diagram synthesizes findings from both approaches, highlighting proteins with consistent evidence across observational and causal inference analyses. The intersecting region contains proteins demonstrating both significant associations and causal relationships, with their identities explicitly labeled. FDR = false discovery rate.

Through comprehensive multivariable Cox regression modeling, incorporating adjustments for demographic characteristics (age, gender, ethnicity), anthropometric measures (body mass index), socioeconomic indicators (household income, deprivation index), lifestyle factors (smoking status, alcohol consumption, physical activity), and temporal variables (seasonality of sample collection), we identified 23 plasma proteins demonstrating significant associations with AD development. These associations maintained statistical significance following stringent FDR correction (FDR < 0.05), as illustrated in Figure 2 and comprehensively documented in eTable 4, Supplemental Digital Content, https://links.lww.com/MD/P444.

### 3.2. Genetic associations of proteins with AD

**Figure 3.**
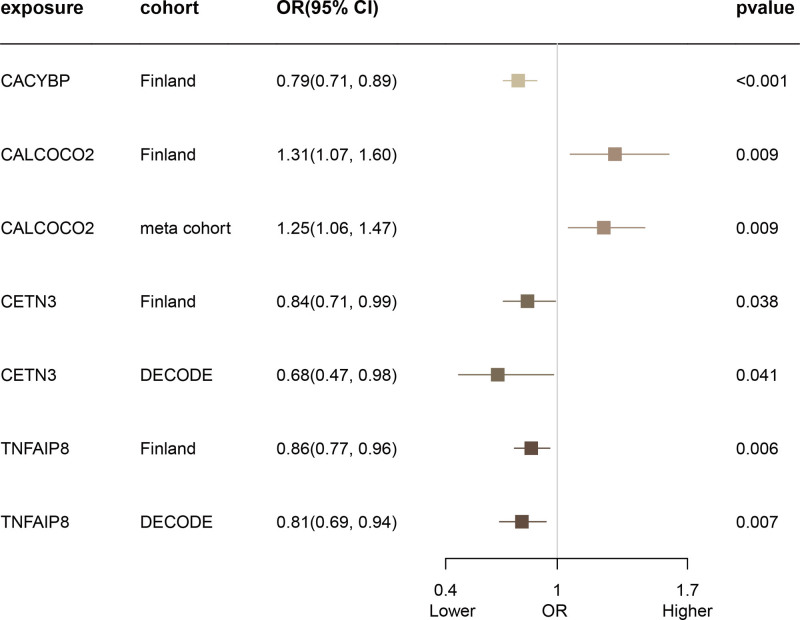
Validating candidate protein causality for atopic dermatitis in 3 additional pQTL databases. pQTL = protein quantitative trait loci.

Subsequent cis-MR analysis evaluated the causal effects of identified proteins on AD. Of the 2920 proteins studied in the UKB-PPP, 2030 cis-pQTLs were identified, as detailed in eTable 5, Supplemental Digital Content, https://links.lww.com/MD/P444. Among the 23 proteins highlighted by the Cox regression analysis, 20 had cis-pQTLs with F statistics exceeding 10, confirming their robustness as instruments. Through MR analysis, 10 of these proteins were causally implicated in AD, as shown in Figure 2 and eTable 6, Supplemental Digital Content, https://links.lww.com/MD/P444. Of these, 9 proteins – CACYBP, CALCOCO2, CETN3, MOCS2, PSTPIP2, PVALB, TARBP2, TNFAIP8, and TNIPI – were identified as protective, while PRRT3 was found to contribute adversely to AD. Steiger filtering confirmed the directional correctness of these associations (eTable 6, Supplemental Digital Content, https://links.lww.com/MD/P444). Phenotypic analysis of significant cis-pQTLs linked to these proteins revealed no associations with known AD risk factors such as BMI, body fat percentage, or smoking habits (eTable 7, Supplemental Digital Content, https://links.lww.com/MD/P444). We also cross-validated the effects of 4 proteins using 3 additional pQTL databases, which confirmed the initial findings for all but CALCOCO2, which exhibited a contrary effect as shown in Figure 3.

Summary-data-based MR analysis indicated significant causal relationships between the expression levels of 5 proteins and AD, excluding CTSE, RPA2, and MMP1 from this cohort. Further scrutiny via the HEIDI test excluded AGER, SEPTIN8, CALCOCO2, and TARBP2 due to indications of horizontal pleiotropy, as detailed in eTable 8, Supplemental Digital Content, https://links.lww.com/MD/P444. Notably, CACYBP demonstrated strong genetic co-localization with a posterior probability for hypothesis 4 of 0.92, suggesting a high likelihood of shared causal genetic variants between CACYBP protein levels and AD risk. This finding is documented in eFigure 2 and eTable 9, Supplemental Digital Content, https://links.lww.com/MD/P444, highlighting the robustness of the genetic association.

### 3.3. Phenome-wide MR analysis

Acknowledging the influential role that proteins play through the bloodstream in pharmacological interventions, our study expanded its focus to examine the effects of 5 specific blood protein expressions on a broad spectrum of health conditions. We undertook a detailed MR analysis that covered 1402 conditions and traits recorded in the UK Biobank. Despite the extensive nature of this analysis, our results showed no significant associations between the protein expressions and the vast array of health conditions explored. These findings are thoroughly detailed in eFigures 3, Supplemental Digital Content, https://links.lww.com/MD/P444.

### 3.4. Differential expression analysis and cell-type specificity expression in the skin tissue

The differential analysis of protein groups targeting specific dermatitis biomarkers revealed varied expression levels. Notably, CALCOCO2 and TARBP2 did not show significant differences in expression levels. However, except for PRRT3, the other 8 targeted proteins were notably overexpressed in the serum of patients who were not diagnosed with specific dermatitis, as detailed in eFigure 4, Supplemental Digital Content, https://links.lww.com/MD/P444.

**Figure 4.**
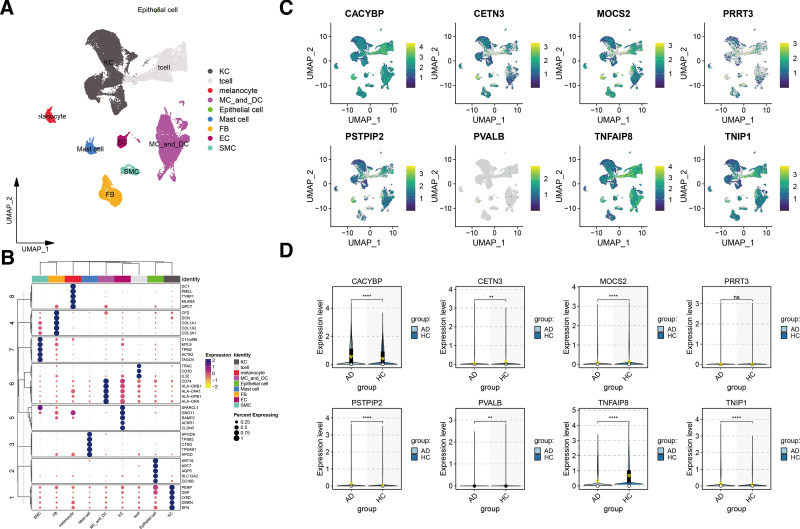
Single-cell resolution analysis of atopic dermatitis molecular signatures. (A) Cellular landscape visualization: the uniform manifold approximation and projection (UMAP) plot illustrates the cellular heterogeneity within atopic dermatitis lesions, with each data point representing an individual cell colored according to its cluster assignment. This dimensionality reduction technique reveals the complex cellular architecture of affected tissue. (B) Cellular identity mapping: a comprehensive heatmap displays cluster-defining marker gene expression patterns, where color intensity and circle diameter represent expression levels and detection frequency, respectively. Key cellular populations are annotated, including fibroblasts (FB), keratinocytes (KC), endothelial cells (EC), macrophages (MC), smooth muscle cells (SMC), and dendritic cells (DC). (C) Gene expression topography: feature plots depict the spatial distribution of 8 AD-associated genes across cellular clusters. Gene expression gradients are represented by a color continuum from dark (high expression) to light (low expression), with each point corresponding to a single cell. (D) Quantitative expression profiling: violin plots provide statistical visualization of differential gene expression between AD and control samples. The plots display probability density distributions of expression values, with yellow inverted triangles marking mean expression levels for each gene across conditions. AD = atopic dermatitis.

To investigate whether the genes encoding these 8 circulating proteins exhibit cell type-specific enrichment in dermatological tissues, we performed a single-cell type expression analysis using scRNA-seq data sourced from the Gene Expression Omnibus. Our analysis of the AD dataset GSE153760 identified 9 distinct cell types, as shown in Figure 4A. The specific expression markers for each cell type are presented in Figure 4B. Notably, proteins such as CACYBP, MOCS2, PSTPIP2, TNFAIP8, and TNIP1 were found to be expressed across various skin cells but exhibited heightened levels in immune cells, including T cells, monocytes, macrophages, and mast cells. The expression of CETN3 and PRRT3 was relatively uniform across all examined cell types, while PVALB was minimally expressed or absent in the cell types analyzed. Further RNA-Seq differential analysis revealed significant expression differences between normal and AD skin tissues. Specifically, CACYBP, PSTPIP2, and TNIP1 showed significantly higher expression in AD tissues. Conversely, CETN3, MOCS2, PVALB, and TNFAIP8 displayed significantly lower expression in AD tissues. The expression of PRRT3, on the other hand, did not exhibit any significant difference between the 2 tissue types.

### 3.5. Identification and stratification of biomarkers

The investigation implemented a hierarchical evidence-based framework for biomarker classification, incorporating multiple orthogonal validation approaches. This stratification system comprises 3 distinct evidentiary levels (Table 2). Evaluation of therapeutic potential through comprehensive drug target analysis revealed that none of the identified biomarker proteins currently serve as molecular targets in clinical drug development pipelines (Table 2).

**Table 2:** Comprehensive evidence to identify biomarkers in atopic dermatitis.

Protein	Incident atopic dermatitis											
	Cohort analyze		Genetic analyze				Expression		Drug development			
	HR (95% CI)	*P*-value/FDR	TSMR		SMR	Coloc	ScRNA-seq & serum protein	Tier	Drug name	Outcomes	Actions	Trial phase
			OR (95% CI)	*P*-value								
CACYBP	0.88 [0.80;0.97]	1.04e−05/0.00793	0.69 [0.56;0.86]	.00074	√	√	√	3	/	/	/	/
CALCOCO2	0.90 [0.81;1.00]	4.44e−05/0.00855	0.58 [0.38;0.89]	.01182	×	×	×	1				
CETN3	0.88 [0.80;0.97]	1.41e−05/0.00793	0.84 [0.71;0.99]	.04091	√	×	√	3	/	/	/	/
MOCS2	0.88 [0.79;0.97]	1.23e−05/0.00793	0.93 [0.88;0.98]	.00709	√	×	√	3	/	/	/	/
PRRT3	1.11 [1.00;1.23]	4.50e−05/0.00855	1.22 [1.05;1.42]	.00951	√	×	×	2	/	/	/	/
PSTPIP2	0.88 [0.80;0.97]	8.93e−05/0.01377	0.84 [0.72;0.99]	.03777	√	×	×	2	/	/	/	/
PVALB	0.90 [0.82;0.99]	3.70e−05/0.00855	0.96 [0.93;1.00]	.04091	√	×	√	3	Formic acid	/	/	I
TARBP2	0.90 [0.82;1.00]	4.67e−05/0.00855	0.46 [0.27;0.81]	.00647	×	×	×	1				
TNFAIP8	0.87 [0.79;0.97]	1.09e−05/0.00793	0.66 [0.49;0.89]	.00692	√	×	√	3	/	/	/	/
TNIP1	0.86 [0.78;0.96]	4.42e−06/0.00793	0.43 [0.24;0.76]	.00362	√	×	×	2	/	/	/	/

CI = confidence interval, FDR = false discovery rate, HR = hard ratio, OR = odds ratios, SMR = summary-data-based MR, TSMR = two-sample Mendelian randomization.

* HR was adjusted by BMI (kilograms per square meter, continuous), age (sequential), sex (male or female), Townsend Deprivation Index (continuous), average annual household gross income (<£18,000, £18,000–£30,999, £31,000–£51,999, £52,000–£100,000, >£100,000, And “not known” or missing), education (CSEs or equivalent, A levels/AS levels or equivalent, College or University degree, NVQ or HND or HNC or equivalent, O levels/GCSEs or equivalent, professional qualifications, and none of the above), the season of blood collection, smoking status (current, previous or never), alcohol consumption (daily, month to week or never), and physical activity (met-minutes per week, Consecutive).

### 3.6. Development of a protein scoring system for AD

**Figure 5.**
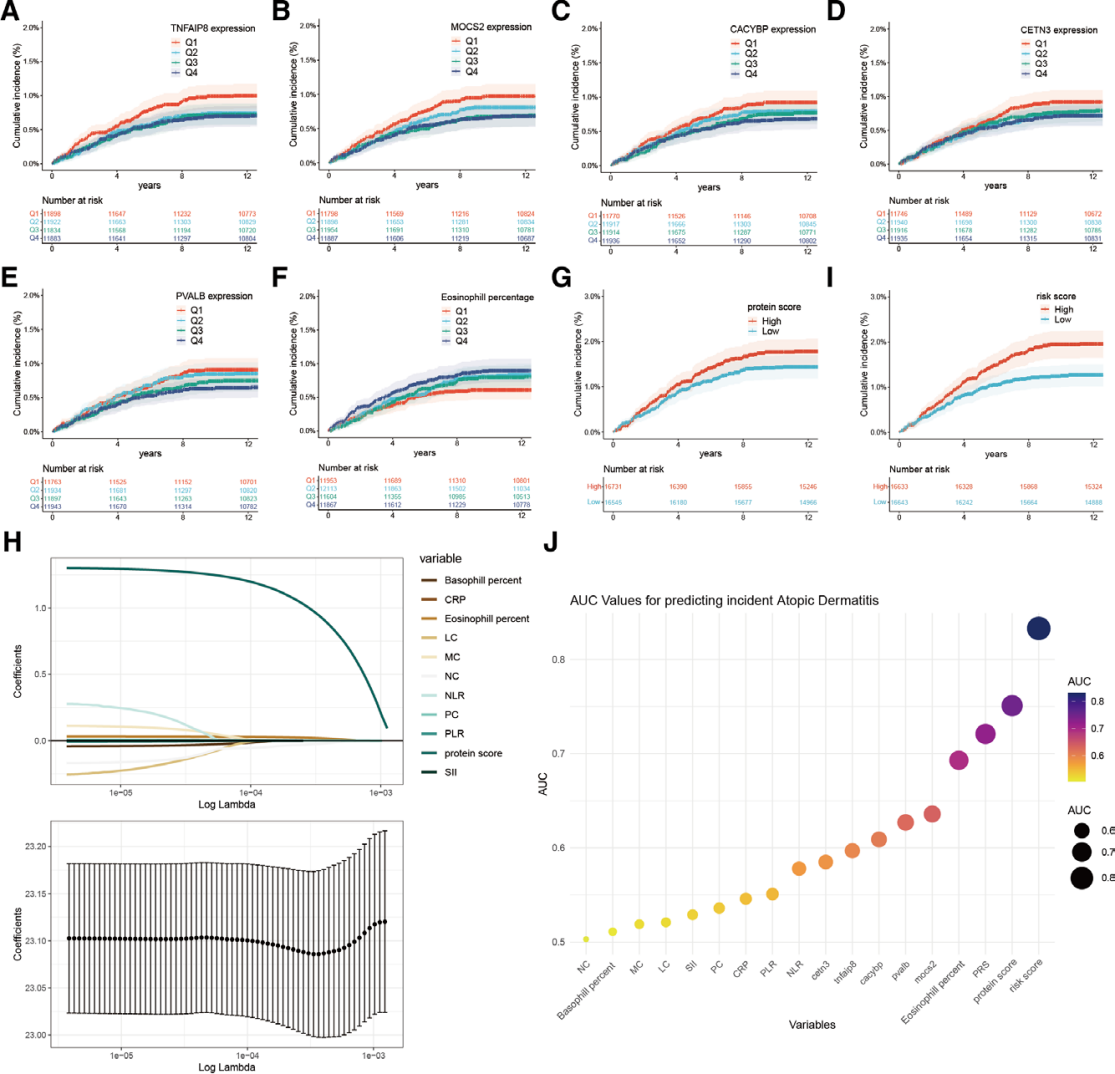
Development of a protein scoring system for atopic dermatitis. (A–G, I) Results of cumulative risk curve analysis of TNFAIP8, MOCS2, CACYBP, CETN3, PVALB, Eosinophil percentage, protein score, and risk score. (H) Results of Lasso regression analysis of multiple variables, each line representing a variable. (J) ROC curves of 5 protein markers, protein scores, risk score, and multiple systemic inflammatory markers for predicting the onset of atopic dermatitis in the train cohort.

The expression levels of 5 biomarker proteins and the eosinophil percentage were classified into quartiles, and cumulative risk curve analysis was conducted. The results indicated that, with the exception of CETN3, increases in the expression levels of the other 4 proteins were significantly associated with a decrease in both the incidence and risk of AD (Fig. 5A–E). In contrast, elevated eosinophil levels were linked to a higher risk of AD (Fig. 5F).

Building on these findings, a protein score was established using multivariate Cox regression analysis, incorporating the 5 proteins. This score showed that individuals with higher scores faced a considerably greater incidence and risk of AD than those with lower scores, both in the training and test cohorts (Fig. 5G and eFigure 5A, Supplemental Digital Content, https://links.lww.com/MD/P444). The AUC for predicting the 5-year incidence of AD using the protein score was 0.751 in the training cohort and 0.763 in the test cohort (Fig. 5J and eFigure 5C, Supplemental Digital Content, https://links.lww.com/MD/P444). These AUC values surpassed those of the PRS and eosinophil percentage.

To further refine the prediction accuracy, lasso regression was applied to 10 inflammatory and immune markers alongside the protein score. Using a lambda value of 4 (standard lambda.1se, Fig. 5H), 4 key molecules (protein score, neutrophil count, platelet count, and eosinophil percentage) were selected for the creation of a risk score. Multivariate Cox regression analysis of these 4 factors resulted in the development of the risk score. Individuals with higher risk scores showed a significantly increased incidence and risk of AD compared to those with lower scores in both the training and test cohorts (Fig. 5I and eFigure 5B, Supplemental Digital Content, https://links.lww.com/MD/P444). The AUC values for predicting the 5-year incidence of AD using the risk score were 0.833 in the training cohort and 0.846 in the test cohort (Fig. 5J and eFigure 5C, Supplemental Digital Content, https://links.lww.com/MD/P444).

## 4. Discussion

Leveraging the extensive UK Biobank Plasma Profiling Project (UKB-PPP) dataset encompassing 51,068 participants and 2923 plasma proteins, our investigation systematically integrated proteomic profiling with genome-wide association data to elucidate both longitudinal associations and causal mechanisms underlying AD pathogenesis. Through this multi-omics approach, we identified 5 plasma proteins with substantial potential as therapeutic targets for AD intervention. Building upon these discoveries, we established 2 novel predictive tools: (1) a protein-based scoring system and (2) an integrated risk assessment model, both incorporating the identified protein biomarkers. These tools demonstrated robust predictive performance for AD onset, offering significant advancements in disease risk stratification. The identification of these protein signatures and the development of corresponding predictive models underscore their potential utility as both diagnostic biomarkers and therapeutic targets in AD clinical management.

The study by Janna et al^[23]^ reported that the transcriptome expression levels of CETN3 (Centrin 3) and MOCS2 (Molybdenum Cofactor Synthesis 2) in AD patients were significantly lower compared to those in the normal control group, findings that align with our own research. Our study corroborates these results, demonstrating decreased expression levels of CETN3 and MOCS2 in AD patients. Furthermore, our cohort analysis revealed that each 1 SD increase in the blood levels of CETN3 and MOCS2 proteins corresponded to a 12% decrease in the incidence of AD, respectively.

The relationship between CACYBP (Calcyclin Binding Protein), PVALB (Parvalbumin), TNFAIP8 (TNF Alpha Induced Protein 8) and AD has been less explored in existing literature. Our findings contribute novel insights, indicating that the expression levels of these 3 molecules in AD patients are also lower than those in the control group, across both hemoglobin groups and skin lesion transcriptomes. The cohort analysis further suggests a protective effect against AD, with the incidence of AD decreasing by 12%, 10%, and 13% for each SD increase in the blood levels of CACYBP, PVALB, and TNFAIP8 proteins, respectively. These results highlight the potential of CETN3, MOCS2, CACYBP, PVALB, and TNFAIP8 as biomarkers for AD, suggesting their downregulation is associated with the disease’s pathology.

AD is a chronic inflammatory skin disease characterized by elevated levels of inflammatory biomarkers, including eosinophils and CRP, which have been proposed as potential predictors of AD onset and severity.^[24–26]^ In this study, we identified 5 novel protein biomarkers for AD and developed a scoring system based on their expression levels. Our findings indicate that higher protein scores are significantly associated with an increased risk of AD. Furthermore, our protein score demonstrated superior predictive capabilities compared to PRS and traditional inflammatory biomarkers such as eosinophils percentage and CRP. Additionally, we constructed a risk score by integrating protein scores with inflammatory indicators, such as the eosinophil ratio. This combined score showed enhanced sensitivity and accuracy in predicting AD. Protein-based scoring systems, similar to those used in cardiovascular disease prediction,^[27,28]^ reflect ongoing biological processes. Our results suggest that this novel evaluation system could significantly improve the prediction of AD onset and prognosis by incorporating a broader spectrum of AD-related proteins.

The methodological strength of this investigation lies in its innovative integration of proteomic profiling with genomic association data, enabling systematic exploration of plasma protein-AD relationships. Nevertheless, several limitations warrant consideration to properly contextualize the study’s implications. Firstly, The predominantly Caucasian composition of our cohort (92.4%) may restrict the generalizability of findings to other ethnic populations, necessitating validation in more diverse cohorts.Secondly, While our analysis focused on circulating proteins, the absence of direct protein measurements from cutaneous lesions represents a notable limitation. Although we employed single-cell transcriptomic data from lesional tissue as a complementary approach, direct proteomic analysis of affected skin would provide more definitive mechanistic insights. Thirdly, the availability of robust cis-pQTLs varied across identified proteins, limiting the scope of comprehensive cis-MR analyses. This constraint, coupled with the variable strength of genetic instruments (particularly those with limited variant numbers), may have resulted in conservative effect estimates. Finally, the lack of serum IgE measurements in our dataset prevented direct comparison of our proteomic risk score with this established AD biomarker. Future investigations incorporating IgE quantification would enable comprehensive evaluation of relative predictive capacities.

## 5. Conclusion

Utilizing the extensive UK Biobank-PPP cohort and employing advanced methods in MR and proteome analysis, our study has successfully identified 5 novel biomarkers for AD. In addition, we developed a protein scoring system that effectively predicts the incidence and risk associated with this condition. Collectively, our findings contribute valuable insights into the complex etiology and progression of AD, potentially revolutionizing its management and treatment.

## Acknowledgments

We extend our deepest gratitude to the numerous researchers who have generously shared their Genome-Wide Association Study (GWAS) datasets, which have been instrumental in the execution of this study.

## Author contributions

**Conceptualization:** Rui Mao.

**Data curation:** Miaoyi Zhang, Tongtong Zhang, Rui Mao.

**Formal analysis:** Sui Deng, Rui Mao.

**Funding acquisition:** Rui Mao.

**Investigation:** Miaoyi Zhang.

**Methodology:** Sui Deng, Rui Mao.

**Project administration:** Miaoyi Zhang, Tongtong Zhang, Rui Mao.

**Resources:** Miaoyi Zhang, Tongtong Zhang.

**Software:** Rui Mao.

**Supervision:** Tongtong Zhang.

**Validation:** Sui Deng, Rui Mao.

**Visualization:** Sui Deng, Rui Mao.

**Writing – original draft:** Sui Deng, Rui Mao.

**Writing – review & editing:** Tongtong Zhang.

## Supplementary Material


