# Supplementary material for: Associations of 2923 plasma proteins with incident atopic dermatitis in a prospective cohort study and genetic analysis
Source: Medicine (Baltimore). 2025 Jul 18;104(29):e43447. doi: 10.1097/MD.0000000000043447 (PMC12282691; doi:10.1097/MD.0000000000043447)
Supplement: Supplementary file 1 [file medi-104-e43447-s001.docx]

Supplement 1

EMethods

eTable 1. STROBE-MR checklist of recommended items to address in reports of Mendelian randomisation studies

eTable 2. Details of the source data.

eTable 3. Details of the single cell RNAseq data.

eTable 4. Associations of circulating protein levels with incident Atopic dermatitis

eTable 5. CIS-pQTL available in UKB-PPP cohort database

eTable 6. Associations between genetically proxied protein expression and Atopic dermatitis

eTable 7. The confuder traits associated with Variants of candidate drugtarget protein.

eTable 8. SMR Associations of Serum Protein Expression with Atopic dermatitis.

eTable 9. Coloc colocalization analysis

eFigure 1. The Theoretical Underpinnings and Principal Assumptions of Mendelian Randomization.

eFigure 2. Bayesian Colocalization Analysis to Unveil Potential Causal Links Between Specific Plasma Proteins and Atopic Dermatitis.

eFigure 3. Phenome-Wide Mendelian Randomization Analysis: Evaluating the Effects of Atopic Dermatitis-Associated Drug Targets Across Diverse Diseases.

eFigure 4. Comparative Serum Protein Expression Analysis of Drug Targets in Atopic Dermatitis.

eFigure 5: Validation of Protein Scoring System for Atopic Dermatitis in the test set

**Emethods**

**Selection of genetic instruments**

The establishment of valid genetic instruments in our analysis required rigorous adherence to three fundamental assumptions, as visually represented in Supplement 1's eFigure 1. Initially, we ensured a robust association between genetic variants and the exposure variable by computing the F-statistic, calculated as the square of the regression coefficient divided by the variance of its standard error. Genetic instruments with F-statistics exceeding 10 were retained to mitigate weak instrument bias. Secondly, we implemented stringent controls to prevent confounding through pleiotropic pathways. Utilizing the PhenoScanner genomic database[1], we systematically evaluated potential associations between genetic variants and AD-related phenotypes, applying a stringent genome-wide significance threshold (P < 5×10^-8^). This screening process identified and excluded variants associated with established confounders including adiposity measures[2], hematological parameters (eosinophil, basophil, and lymphocyte counts)[3], and rheumatoid arthritis[4], thereby reducing horizontal pleiotropy concerns. Thirdly, we implemented multiple strategies to ensure the exclusion restriction assumption: (1) restricting analyses to individuals of European ancestry to minimize population stratification effects, and (2) conducting summary-data-based Mendelian randomization (SMR) and heterogeneity in dependent instruments (HEIDI) analyses to address potential linkage disequilibrium confounding.

***Bayesian co-localization analysis***

To evaluate whether two traits—protein expression levels and AD—are influenced by identical causal genetic variants, we employed Bayesian co-localization analysis using the 'coloc' package[5], accessible at https://github.com/chr1swallace/coloc. This method calculates posterior probabilities for five distinct hypotheses regarding the potential overlap of genetic variants between the proteome and AD. Our analysis particularly focused on the posterior probability of hypothesis 3 (PPH3) and hypothesis 4 (PPH4). PPH3 suggests that the protein expression and ISD are influenced by separate genetic variants within the same region, while PPH4 indicates a shared genetic basis for both traits in the region. To determine evidence of co-localization, we utilized the coloc.abf and coloc.susie algorithms. A gene was considered to show co-localization if it demonstrated a gene-based PPH4 greater than 80%, as confirmed by at least one of the algorithms.

***SMR analysis and Heterogeneity in Dependent Instruments (HEIDI) test***

In employing pQTL for research, the SMR method is utilized to derive effect estimates. This approach capitalizes on aggregated data from Genome-Wide Association Studies (GWAS) and pQTL studies to investigate the relationships between protein expression levels and specific health outcomes[6]. Analysis and allele coordination were executed using the SMR software, version 1.3.1, available at [https://yanglab.westlake.edu.cn/software/smr/]. To amalgamate the effect estimates, an IVW-MR strategy was adopted. The HEIDI test, integral to the SMR framework, was employed to determine if the gene expression-outcome associations could be attributed to linkage scenarios. This is discerned when the HEIDI test produces a p-value of less than 0.01, suggesting a possible linkage origin for the association[7].

***Phenome-Wide MR Analysis***

To investigate potential adverse reactions associated with the target druggable gene, we conducted phenome-wide MR analyses. In this process, pQTL data related to the druggable gene served as the exposure variable. This data was contrasted with comprehensive GWAS data on a variety of diseases documented within the UK Biobank cohort (n ≤ 408,961), which acted as the outcome variable. Lee et al. have performed GWAS analyses on multiple diseases within the UK Biobank, utilizing the SAIGE (V.0.29) methodology[8,9]. This approach, known for its adept extension and accurate application of generalized mixed models, effectively navigates the complexities of imbalanced case-control ratios. For our phenome-wide MR explorations, we focused on 1,402 traits, each representing a unique disease with no fewer than 50 reported cases. We sourced composite statistics for disease-associated SNPs from the SAIGE GWAS database (available at: https://www.leelabsg.org/resources), with further methodological details outlined in their publication. The Wald ratio method was applied to perform MR analyses, ensuring a uniform approach to parameter application. A stringent significance threshold of P < 0.05/1,402 was set to identify causally significant associations.

***Single-cell RNA Sequencing Data Analysis***

Single-cell RNA sequencing (scRNA-seq) datasets were sourced from publicly accessible databases[10]. The specifics of dataset, including their origins and characteristics, are detailed in eTable 4 of **supplement 1**. For the analysis of the datasets, we employed the Seurat[11] software package, initiating the process with stringent quality control criteria. This involved selecting cells that exhibited a range of nFeature counts from 200 to 5000 and contained less than 25% mitochondrial RNA content, ensuring the exclusion of low-quality or dying cells. Following quality control, dataset integration was performed using the Harmony package, a method chosen for its efficacy in harmonizing data from different sources while retaining biological variability. The integrated datasets then underwent normalization to correct for technical variations, and dimensionality was reduced using Unified Manifold Approximation and Projection (UMAP). Subsequent clustering allowed for the categorization of cells into groups based on their gene expression profiles. These groups were then annotated with cell-specific markers, enabling the identification of distinct cell populations within the datasets.

**The Polygenic Risk Score methods**

The Polygenic Risk Score (PRS) was computed utilizing data from the largest genome-wide association study (GWAS) on AD to date[12]. The genotyping protocols, data imputation processes, and quality control measures applied to the UK Biobank data are thoroughly documented in previous studies[13,14]. Briefly, single nucleotide polymorphisms (SNPs) with minor allele frequencies (MAF) below 1% or with low informativeness (information scores below 0.8) were excluded. Additionally, SNPs that were mismatched, duplicated, or ambiguous were also omitted. The PRS was calculated for each participant using the PRSice-2 software, version 2.3.5, which integrated the UK Biobank SNP database with GWAS summary statistics, and adjusted for sex and ten principal genetic components.

**Drug target analysis**

Biomarker classification followed established pharmacological categorization frameworks, distinguishing between therapeutic clinical applications (Tclin) and chemical characteristics (Tchem). Comprehensive biomarker evaluations, including their therapeutic development stages (phase II-IV clinical trials), were systematically extracted from authoritative pharmacological repositories: DrugBank[15] and OpenTargets databases.

***References***

1 Kamat MA, Blackshaw JA, Young R, *et al.* PhenoScanner V2: an expanded tool for searching human genotype-phenotype associations. *Bioinformatics*. 2019;35:4851–3. doi: 10.1093/bioinformatics/btz469

2 Pan CX, Jee Y-H, Moore KJ, *et al.* Relationship between body mass index and atopic dermatitis: a Mendelian randomization approach in exploring causality: a critical appraisal. *Br J Dermatol*. 2021;184:1045–6. doi: 10.1111/bjd.19702

3 Zeng-Yun-Ou Z, Zhong-Yu J, Wei L. Bidirectional associations between eosinophils, basophils, and lymphocytes with atopic dermatitis: A multivariable Mendelian randomization study. *Front Immunol*. 2022;13:1001911. doi: 10.3389/fimmu.2022.1001911

4 Zhou W, Cai J, Li Z, *et al.* Association of atopic dermatitis with autoimmune diseases: A bidirectional and multivariable two-sample mendelian randomization study. *Front Immunol*. 2023;14:1132719. doi: 10.3389/fimmu.2023.1132719

5 Wang G, Sarkar A, Carbonetto P, *et al.* A simple new approach to variable selection in regression, with application to genetic fine mapping. *J R Stat Soc Series B Stat Methodol*. 2020;82:1273–300. doi: 10.1111/rssb.12388

6 Zhu Z, Zhang F, Hu H, *et al.* Integration of summary data from GWAS and eQTL studies predicts complex trait gene targets. *Nat Genet*. 2016;48:481–7. doi: 10.1038/ng.3538

7 Chauquet S, Zhu Z, O’Donovan MC, *et al.* Association of Antihypertensive Drug Target Genes With Psychiatric Disorders: A Mendelian Randomization Study. *JAMA Psychiatry*. 2021;78:623–31. doi: 10.1001/jamapsychiatry.2021.0005

8 Zhou W, Nielsen JB, Fritsche LG, *et al.* Efficiently controlling for case-control imbalance and sample relatedness in large-scale genetic association studies. *Nat Genet*. 2018;50:1335–41. doi: 10.1038/s41588-018-0184-y

9 Zhou W, Bi W, Zhao Z, *et al.* SAIGE-GENE+ improves the efficiency and accuracy of set-based rare variant association tests. *Nat Genet*. 2022;54:1466–9. doi: 10.1038/s41588-022-01178-w

10 Rojahn TB, Vorstandlechner V, Krausgruber T, *et al.* Single-cell transcriptomics combined with interstitial fluid proteomics defines cell type-specific immune regulation in atopic dermatitis. *J Allergy Clin Immunol*. 2020;146:1056–69. doi: 10.1016/j.jaci.2020.03.041

11 Hao Y, Hao S, Andersen-Nissen E, *et al.* Integrated analysis of multimodal single-cell data. *Cell*. 2021;184:3573-3587.e29. doi: 10.1016/j.cell.2021.04.048

12 Budu-Aggrey A, Kilanowski A, Sobczyk MK, *et al.* European and multi-ancestry genome-wide association meta-analysis of atopic dermatitis highlights importance of systemic immune regulation. *Nat Commun*. 2023;14:6172. doi: 10.1038/s41467-023-41180-2

13 Sudlow C, Gallacher J, Allen N, *et al.* UK biobank: an open access resource for identifying the causes of a wide range of complex diseases of middle and old age. *PLoS Med*. 2015;12:e1001779. doi: 10.1371/journal.pmed.1001779

14 Bycroft C, Freeman C, Petkova D, *et al.* The UK Biobank resource with deep phenotyping and genomic data. *Nature*. 2018;562:203–9. doi: 10.1038/s41586-018-0579-z

15 Knox C, Wilson M, Klinger CM, *et al.* DrugBank 6.0: the DrugBank Knowledgebase for 2024. *Nucleic Acids Res*. 2024;52:D1265–75. doi: 10.1093/nar/gkad976

| **eTable 1. STROBE-MR checklist of recommended items to address in reports of Mendelian randomisation studies** | | | |
| --- | --- | --- | --- |
| **Item No.** | **Section** | **Checklist item** | **Section (paragraph number)** |
| 1 | **TITLE and ABSTRACT** | Indicate Mendelian randomisation as the study’s design in the title and/or the abstract if that is a main purpose of the study | Title page & Abstract page |
|  | **INTRODUCTION** |  |  |
| 2 | **Background** | Explain the scientific background and rationale for the reported study. What is the exposure? Is a potential causal relationship between exposure and outcome plausible? Justify why MR is a helpful method to address the study question | Introduction (paragraphs 1-2) |
| 3 | **Objectives** | State specific objectives clearly, including pre-specified causal hypotheses (if any). State that MR is a method that, under specific assumptions, intends to estimate causal effects | Introduction (paragraph 3) & Figure 1 |
|  | **METHODS** |  |  |
| 4 | **Study design and data sources** | Present key elements of the study design early in the article. Consider including a table listing sources of data for all phases of the study. For each data source contributing to the analysis, describe the following: | Methods (Mendelian Randomization analysis & eTable 3) |
|  | a) | Setting: Describe the study design and the underlying population, if possible. Describe the setting, locations, and relevant dates, including periods of recruitment, exposure, follow-up, and data collection, when available. | Methods (Mendelian Randomization analysis & eTable 3) |
|  | b) | Participants: Give the eligibility criteria, and the sources and methods of selection of participants. Report the sample size, and whether any power or sample size calculations were carried out prior to the main analysis | Methods (Mendelian Randomization analysis & eTable 3) |
|  | c) | Describe measurement, quality control and selection of genetic variants | Methods (Mendelian Randomization analysis & eTable 3) |
|  | d) | For each exposure, outcome, and other relevant variables, describe methods of assessment and diagnostic criteria for diseases | Methods (Mendelian Randomization analysis & eTable 3) |
|  | e) | Provide details of ethics committee approval and participant informed consent, if relevant | Methods (Data availability ) |
| 5 | **Assumptions** | Explicitly state the three core IV assumptions for the main analysis (relevance, independence and exclusion restriction) as well assumptions for any additional or sensitivity analysis | Methods (Mendelian randomization analysis) & Emethods (Bayesian co-localization analysis) |
| 6 | **Statistical methods: main analysis** | Describe statistical methods and statistics used |  |
|  | a) | Describe how quantitative variables were handled in the analyses (i.e., scale, units, model) | Methods (Mendelian randomization analysis) |
|  | b) | Describe how genetic variants were handled in the analyses and, if applicable, how their weights were selected | Methods (Mendelian randomization analysis) |
|  | c) | Describe the MR estimator (e.g. two-stage least squares, Wald ratio) and related statistics. Detail the included covariates and, in case of two-sample MR, whether the same covariate set was used for adjustment in the two samples | Methods (Mendelian randomization analysis) |
|  | d) | Explain how missing data were addressed | N/A |
|  | e) | If applicable, indicate how multiple testing was addressed | Methods (Statistical Analysis) |
| 7 | **Assessment of assumptions** | Describe any methods or prior knowledge used to assess the assumptions or justify their validity | Methods (Mendelian randomization analysis) & Emethods (Bayesian co-localization analysis) |
| 8 | **Sensitivity analyses and additional analyses** | Describe any sensitivity analyses or additional analyses performed (e.g. comparison of effect estimates from different approaches, independent replication, bias analytic techniques, validation of instruments, simulations) | Methods (Mendelian randomization analysis) & Emethods (Bayesian co-localization analysis) |
| 9 | **Software and pre-registration** |  |  |
|  | a) | Name statistical software and package(s), including version and settings used | Methods (Statistical analysis) |
|  | b) | State whether the study protocol and details were pre-registered (as well as when and where) | N/A |
|  | **RESULTS** |  |  |
| 10 | **Descriptive data** |  |  |
|  | a) | Report the numbers of individuals at each stage of included studies and reasons for exclusion. Consider use of a flow-diagram | Results (Genetic associations of proteins with conventional inflammatory skin diseases) & eTable 3 |
|  | b) | Report summary statistics for phenotypic exposure(s), outcome(s) and other relevant variables (e.g. means, SDs, proportions) | eTable 3 |
|  | c) | If the data sources include meta-analyses of previous studies, provide the assessments of heterogeneity across these studies | N/A |
|  | d) | For two-sample Mendelian randomisation: | N/A |
|  |  | i.  Provide justification of the similarity of the genetic variant-exposure associations between the exposure and outcome samples |  |
|  |  | ii.  Provide information on the number of individuals who were in both samples for the exposure and for the outcome |  |
| 11 | **Main results** |  |  |
|  | a) | Report the associations between genetic variant and exposure, and between genetic variant and outcome, preferably on an interpretable scale | Results (Genetic associations of proteins with conventional inflammatory skin diseases) & eTable 10 |
|  | b) | Report MR estimates of the relationship between exposure and outcome, and the measures of uncertainty from the MR analysis, on an interpretable scale, such as odds ratio or relative risk per SD difference | Results (Exploring Aging-Related Drug Targets) & eTable 11-15 |
|  | c) | If relevant, consider translating estimates of relative risk into absolute risk for a meaningful time period | N/A |
|  | d) | Consider plots to visualize results (e.g. forest plot, scatterplot of associations between genetic variants and outcome versus between genetic variants and exposure) | Figures 2 |
| 12 | **Assessment of assumptions** |  |  |
|  | a) | Report the assessment of the validity of the assumptions | Results (Genetic associations of proteins with conventional inflammatory skin diseases) |
|  | b) | Report any additional statistics (e.g., assessments of heterogeneity across genetic variants, such as I^2^, Q statistic or E-value) | eTable 10 |
| 13 | **Sensitivity analyses and additional analyses** |  |  |
|  | a) | Report any sensitivity analyses to assess the robustness of the main results to violations of the assumptions | Results (Genetic associations of proteins with conventional inflammatory skin diseases) |
|  | b) | Report results from other sensitivity analyses or additional analyses |  |
|  | c) | Report any assessment of direction of causal relationship (e.g., bidirectional MR) | Results (Genetic associations of proteins with conventional inflammatory skin diseases) |
|  | d) | When relevant, report and compare with estimates from non-MR analyses | N/A |
|  | e) | Consider additional plots to visualize results (e.g., leave-one-out analyses) | Figure 2 |
|  | **DISCUSSION** |  |  |
| 14 | **Key results** | Summarize key results with reference to study objectives | Discussion (paragraph 1) |
| 15 | **Limitations** | Discuss limitations of the study, taking into account the validity of the IV assumptions, other sources of potential bias, and imprecision. Discuss both direction and magnitude of any potential bias and any efforts to address them | Discussion (paragraph 7) |
| 16 | **Interpretation** |  |  |
|  | a) | Meaning: Give a cautious overall interpretation of results in the context of their limitations and in comparison with other studies | Discussion (paragraph 1-6) |
|  | b) | Mechanism: Discuss underlying biological mechanisms that could drive a potential causal relationship between the investigated exposure and the outcome, and whether the gene-environment equivalence assumption is reasonable. Use causal language carefully, clarifying that IV estimates may provide causal effects only under certain assumptions | Discussion (paragraph 2-6) |
|  | c) | Clinical relevance: Discuss whether the results have clinical or public policy relevance, and to what extent they inform effect sizes of possible interventions | Discussion (paragraph 7) |
| 17 | **Generalizability** | Discuss the generalizability of the study results (a) to other populations, (b) across other exposure periods/timings, and (c) across other levels of exposure | Discussion (paragraph 7) |
|  | **OTHER INFORMATION** |  |  |
| 18 | **Funding** | Describe sources of funding and the role of funders in the present study and, if applicable, sources of funding for the databases and original study or studies on which the present study is based | Funding section |
| 19 | **Data and data sharing** | Provide the data used to perform all analyses or report where and how the data can be accessed, and reference these sources in the article. Provide the statistical code needed to reproduce the results in the article,or report whether the code is publicly accessible and if so, where | Data share statement & eTable 3 |
| 20 | **Conflicts of Interest** | All authors should declare all potential conflicts of interest | Competing interests section |

| **eTable 2. Details of the source data.** | | | | | | | | | |
| --- | --- | --- | --- | --- | --- | --- | --- | --- | --- |
| **GWAS ID** | **Year** | **Trait** | **Consortium** | **Sample size** | **Case** | **Control** | **PMID** | **Population** |  |
| Serum protein data | 2023 | 2923 Serum protein expression | UKB-PPP | 53,021 |  |  | 37794186 | European |  |
| Serum cis Pqtl training cohort | 2023 | 2923 Serum protein expression | UKB-PPP | 53,021 |  |  | 37794186 | European |  |
| Serum cis Pqtl validation cohort | 2021 | 4907 Serum protein expression | DECODE | 35,559 |  |  | 34857953 | European |  |
| Serum cis Pqtl validation cohort | 2021 | 4775 Serum protein expression | FinnGen | 10,708 |  |  | 34648354 | European |  |
| Serum cis Pqtl validation cohort | 2020 | 734 Serum protein expression | GWAS_meta analysis |  |  |  | 32895551 | European |  |
| L12_ATOPIC | 2023 | Atopic dermatitis | FinnGen | 382,254 | 15,208 | 367,046 | 36653562 | European |  |

| **eTable 3. Details of the single cell RNAseq data.** | | | | | |
| --- | --- | --- | --- | --- | --- |
| **Trait** | **dataset** | **Sample size** | **Case** | **Control** | **PMID** |
| Atopic dermatitis | GSE153760 | 15 | 8 | 7 | 32344053 |

| **eTable 4. Associations of circulating protein levels with incident Atopic dermatitis** | | | | | | | |
| --- | --- | --- | --- | --- | --- | --- | --- |
| Variable | HR | Lower | Upper | PValue | adjusted_pvalue | Protein.panel | UniProt |
| TNIP1 | 0.86 | 0.78 | 0.96 | 4.42E-06 | 0.008 | Neurology_II | Q15025 |
| CACYBP | 0.88 | 0.80 | 0.97 | 1.04E-05 | 0.008 | Inflammation_II | Q9HB71 |
| MOCS2 | 0.88 | 0.79 | 0.97 | 1.23E-05 | 0.008 | Inflammation_II | O96007 |
| TNFAIP8 | 0.87 | 0.79 | 0.97 | 1.09E-05 | 0.008 | Inflammation | O95379 |
| CETN3 | 0.88 | 0.80 | 0.97 | 1.41E-05 | 0.008 | Oncology_II | O15182 |
| SLMAP | 0.88 | 0.79 | 0.98 | 1.62E-05 | 0.008 | Oncology_II | Q14BN4 |
| IL21R | 1.14 | 1.06 | 1.23 | 3.44E-05 | 0.009 | Inflammation_II | Q9HBE5 |
| NEXN | 0.82 | 0.74 | 0.91 | 2.32E-05 | 0.009 | Inflammation_II | Q0ZGT2 |
| AKT3 | 0.86 | 0.78 | 0.96 | 4.5E-05 | 0.009 | Oncology | Q9Y243 |
| CLIP2 | 0.87 | 0.79 | 0.96 | 4.39E-05 | 0.009 | Inflammation | Q9UDT6 |
| AIF1 | 0.90 | 0.81 | 0.99 | 3.53E-05 | 0.009 | Oncology | P55008 |
| CD2AP | 0.90 | 0.81 | 0.99 | 3.66E-05 | 0.009 | Cardiometabolic | Q9Y5K6 |
| PVALB | 0.90 | 0.82 | 0.99 | 3.7E-05 | 0.009 | Oncology | P20472 |
| CALCOCO2 | 0.90 | 0.81 | 1.00 | 4.44E-05 | 0.009 | Cardiometabolic_II | Q13137 |
| PRRT3 | 1.11 | 1.00 | 1.23 | 4.5E-05 | 0.009 | Cardiometabolic_II | Q5FWE3 |
| TARBP2 | 0.90 | 0.82 | 1.00 | 4.67E-05 | 0.009 | Neurology | Q15633 |
| MAP2K1 | 0.84 | 0.76 | 0.93 | 5.32E-05 | 0.009 | Oncology_II | Q02750 |
| ADH1B | 1.14 | 1.04 | 1.26 | 7.66E-05 | 0.012 | Inflammation_II | P00325 |
| PSTPIP2 | 0.88 | 0.80 | 0.97 | 8.93E-05 | 0.014 | Inflammation_II | Q9H939 |
| CLTA | 0.80 | 0.72 | 0.89 | 9.55E-05 | 0.014 | Cardiometabolic | P09496 |
| COL9A1 | 1.19 | 1.08 | 1.30 | 0.000228 | 0.032 | Inflammation | P20849 |
| FRMD4B | 0.82 | 0.73 | 0.91 | 0.000244 | 0.032 | Cardiometabolic_II | Q9Y2L6 |
| IPCEF1 | 0.83 | 0.76 | 0.92 | 0.000337 | 0.043 | Neurology | Q8WWN9 |

| **eTable 5. CIS-pQTL available in UKB-PPP cohort database** | | | | | | | | | | |
| --- | --- | --- | --- | --- | --- | --- | --- | --- | --- | --- |
| SNP | chr | pos | eaf | beta | se | pval | other_allele | effect_allele | exposure | F_statistics |
| rs2066702 | 4 | 99307860 | 0.007 | -0.922 | 0.040 | 2.64E-115 | G | A | ADH1B | 520.9 |
| rs2261033 | 6 | 31635814 | 0.426 | -0.100 | 0.006 | 4.31E-56 | A | G | AIF1 | 249.0 |
| rs320339 | 1 | 243707608 | 0.197 | 0.070 | 0.008 | 1.62E-18 | G | T | AKT3 | 77.1 |
| rs76088187 | 1 | 174875870 | 0.116 | -0.177 | 0.010 | 3.91E-75 | A | G | CACYBP | 336.4 |
| rs606911 | 17 | 48830866 | 0.158 | 0.073 | 0.008 | 1.14E-18 | G | A | CALCOCO2 | 77.8 |
| rs9463335 | 6 | 47511400 | 0.267 | 0.306 | 0.007 | 1.00E-200 | G | A | CD2AP | 2077.7 |
| rs4873 | 5 | 90407824 | 0.272 | -0.177 | 0.007 | 3.56E-145 | C | G | CETN3 | 658.3 |
| rs73131317 | 7 | 74288385 | 0.065 | 0.231 | 0.012 | 1.96E-77 | G | A | CLIP2 | 346.9 |
| rs543674622 | 6 | 70277423 | 0.003 | 1.474 | 0.061 | 1.89E-130 | A | G | COL9A1 | 590.6 |
| rs883273 | 6 | 154309407 | 0.323 | 0.086 | 0.007 | 2.05E-39 | C | A | IPCEF1 | 172.6 |
| rs6880055 | 5 | 53093598 | 0.338 | 0.455 | 0.006 | 1.00E-200 | A | G | MOCS2 | 4895.4 |
| rs17387365 | 1 | 77972454 | 0.076 | -0.143 | 0.011 | 4.29E-36 | G | T | NEXN | 157.3 |
| rs3846167 | 3 | 9954742 | 0.260 | -0.211 | 0.007 | 1.00E-200 | T | C | PRRT3 | 934.4 |
| rs72642434 | 18 | 45992711 | 0.141 | -0.222 | 0.009 | 7.83E-131 | T | A | PSTPIP2 | 592.3 |
| rs4821544 | 22 | 36862461 | 0.313 | -0.816 | 0.007 | 1.00E-200 | T | C | PVALB | 15000.1 |
| rs1354034 | 3 | 56815721 | 0.589 | 0.072 | 0.006 | 2.05E-31 | T | C | SLMAP | 135.9 |
| rs10876550 | 12 | 54318524 | 0.572 | 0.045 | 0.006 | 5.50E-13 | G | A | TARBP2 | 52.0 |
| rs1035376 | 5 | 119312555 | 0.883 | 0.115 | 0.010 | 5.98E-32 | G | A | TNFAIP8 | 138.4 |
| rs12518386 | 5 | 151058524 | 0.254 | -0.048 | 0.007 | 2.73E-12 | G | A | TNIP1 | 48.9 |
| rs554722848 | 15 | 66365281 | 0.092 | 0.091 | 0.011 | 1.06E-17 | TATACATA | T | MAP2K1 | 73.4 |

| **eTable 6. Associations between genetically proxied protein expression and Atopic dermatitis** | | | | | | | | |
| --- | --- | --- | --- | --- | --- | --- | --- | --- |
| exposure | method | nsnp | pval | pve | or | or_lci95 | or_uci95 | steiger_pval |
| TNIP1 | Wald ratio | 1 | 0.004 | 0.001 | 0.430 | 0.244 | 0.759 | 1.98E-84 |
| CACYBP | Wald ratio | 1 | 0.001 | 0.010 | 0.690 | 0.556 | 0.856 | 2.74E-27 |
| PRRT3 | Wald ratio | 1 | 0.010 | 0.026 | 1.220 | 1.050 | 1.417 | 0.00E+00 |
| CLIP2 | Wald ratio | 1 | 0.846 | 0.010 | 0.982 | 0.816 | 1.181 | 2.92E-21 |
| SLMAP | Wald ratio | 1 | 0.032 | 0.004 | 1.475 | 1.034 | 2.104 | 7.49E-163 |
| AKT3 | Wald ratio | 1 | 0.081 | 0.002 | 0.700 | 0.469 | 1.045 | 3.34E-21 |
| TARBP2 | Wald ratio | 1 | 0.006 | 0.002 | 0.462 | 0.265 | 0.806 | 3.56E-194 |
| PSTPIP2 | Wald ratio | 1 | 0.038 | 0.017 | 0.844 | 0.719 | 0.990 | 0.00E+00 |
| COL9A1 | Wald ratio | 1 | 0.010 | 0.017 | 0.860 | 0.766 | 0.965 | 7.16E-202 |
| TNFAIP8 | Wald ratio | 1 | 0.007 | 0.004 | 0.661 | 0.490 | 0.893 | 1.23E-154 |
| CD2AP | Wald ratio | 1 | 0.013 | 0.057 | 1.127 | 1.025 | 1.239 | 1.28E-31 |
| CETN3 | Wald ratio | 1 | 0.041 | 0.019 | 0.840 | 0.711 | 0.993 | 0.00E+00 |
| NEXN | Wald ratio | 1 | 0.859 | 0.005 | 1.021 | 0.809 | 1.289 | 2.24E-241 |
| MOCS2 | Wald ratio | 1 | 0.007 | 0.124 | 0.930 | 0.881 | 0.980 | 1.12E-255 |
| IPCEF1 | Wald ratio | 1 | 0.076 | 0.005 | 0.776 | 0.587 | 1.026 | 1.72E-280 |
| AIF1 | Wald ratio | 1 | 0.040 | 0.007 | 1.284 | 1.012 | 1.631 | 3.59E-46 |
| CALCOCO2 | Wald ratio | 1 | 0.012 | 0.002 | 0.579 | 0.379 | 0.886 | 2.23E-50 |
| PVALB | Wald ratio | 1 | 0.041 | 0.303 | 0.964 | 0.931 | 0.998 | 0.00E+00 |
| TNIP1 | Wald ratio | 1 | 0.004 | 0.001 | 0.430 | 0.244 | 0.759 | 1.98E-84 |
| CACYBP | Wald ratio | 1 | 0.001 | 0.010 | 0.690 | 0.556 | 0.856 | 2.74E-27 |

| **eTable 7. The confuder traits associated with Variants of candidate drugtarget protein.** | | | | | | |
| --- | --- | --- | --- | --- | --- | --- |
| exposure | outcome | snp | r2 | trait | p | catalog |
| TARBP2 | Atopic dermatitis | rs10876550 | 1 | Red blood cell count | 1.54E-10 | GWAS |
| TARBP2 | Atopic dermatitis | rs10876550 | 1 | Mean platelet volume | 2.00E-14 | GWAS |
| TARBP2 | Atopic dermatitis | rs10876550 | 1 | Mean platelet volume | 1.81E-152 | GWAS |
| TARBP2 | Atopic dermatitis | rs10876550 | 0.98012 | Platelet distribution width | 6.97E-42 | GWAS |
| TARBP2 | Atopic dermatitis | rs10876550 | 1 | Mean platelet volume MPV | 1.86E-14 | GWAS |
| TARBP2 | Atopic dermatitis | rs10876550 | 0.98012 | C-C motif chemokine 28 | 1.91E-14 | pQTL |
| TARBP2 | Atopic dermatitis | rs10876550 | 0.93302 | Platelet count | 4.57E-08 | GWAS |
| TARBP2 | Atopic dermatitis | rs10876550 | 0.93302 | Platelet counts | 5.00E-08 | GWAS |
| TARBP2 | Atopic dermatitis | rs10876550 | 0.98012 | Platelet count | 2.00E-54 | GWAS |
| PRRT3 | Atopic dermatitis | rs3846167 | 1 | Cysteine-rich with EGF-like domain protein 1 | 0 | pQTL |
| PRRT3 | Atopic dermatitis | rs3846167 | 0.99407 | Cysteine-rich with EGF-like domain protein 1 | 0 | pQTL |
| PRRT3 | Atopic dermatitis | rs3846167 | 0.99407 | Cysteine-rich with EGF-like domain protein 1 | 0 | pQTL |
| PRRT3 | Atopic dermatitis | rs3846167 | 0.99407 | Cysteine-rich with EGF-like domain protein 1 | 0 | pQTL |
| PRRT3 | Atopic dermatitis | rs3846167 | 1 | Cysteine-rich with EGF-like domain protein 1 | 0 | pQTL |
| PVALB | Atopic dermatitis | rs4821544 | 1 | Eosinophil counts | 5.00E-13 | GWAS |
| PVALB | Atopic dermatitis | rs4821544 | 1 | Sum eosinophil basophil counts | 5.00E-14 | GWAS |
| PVALB | Atopic dermatitis | rs4821544 | 1 | Eosinophil percentage of white cells | 7.37E-11 | GWAS |
| PVALB | Atopic dermatitis | rs4821544 | 1 | Crohns disease | 2.00E-08 | GWAS |
| PVALB | Atopic dermatitis | rs4821544 | 1 | Eosinophil count | 4.98E-13 | GWAS |
| PVALB | Atopic dermatitis | rs4821544 | 1 | Eosinophil percentage of granulocytes | 1.58E-09 | GWAS |
| PVALB | Atopic dermatitis | rs4821544 | 1 | Neutrophil percentage of granulocytes | 2.54E-09 | GWAS |

| **eTable 8.** SMR Associations of Serum Protein Expression with Atopic dermatitis. | | | | | | | | | | | | |
| --- | --- | --- | --- | --- | --- | --- | --- | --- | --- | --- | --- | --- |
| **Gene** | **top SNP** | **pQTL association** | | | **GWAS association** | | | **SMR association** | | | **HEIDI Test** | |
|  |  | **beta** | **se** | **p-value** | **beta** | **se** | **p-value** | **beta** | **se** | **p-value** | **p-value** | **Number of SNPs** |
| TNIP1* | \ | \ | \ | \ | \ | \ | \ | \ | \ | \ | \ | \ |
| CACYBP | rs12565973 | -0.171 | 0.012 | 7.89E-46 | 0.087 | 0.020 | 9.69E-06 | -0.507 | 0.120 | 2.40E-05 | 1.48E-01 | 20 |
| **CALCOCO2** | **rs606911** | **0.075** | **0.010** | **1.37E-13** | **-0.040** | **0.016** | **1.18E-02** | **-0.532** | **0.223** | **1.72E-02** | **5.57E-04** | **20** |
| CETN3 | rs4873 | -0.173 | 0.008 | 3.98E-93 | 0.031 | 0.015 | 4.09E-02 | -0.178 | 0.087 | 4.19E-02 | 8.95E-01 | 20 |
| MOCS2 | rs6880055 | 0.449 | 0.008 | 0.00E+00 | -0.033 | 0.012 | 7.09E-03 | -0.074 | 0.028 | 7.15E-03 | 8.35E-01 | 20 |
| PRRT3 | rs55847233 | -0.213 | 0.009 | 1.14E-132 | -0.042 | 0.016 | 9.48E-03 | 0.197 | 0.076 | 9.89E-03 | 3.04E-01 | 20 |
| PSTPIP2 | rs72642432 | -0.226 | 0.011 | 1.93E-87 | 0.038 | 0.018 | 3.77E-02 | -0.167 | 0.081 | 3.88E-02 | 7.96E-02 | 20 |
| PVALB | rs4821544 | -0.813 | 0.008 | 0.00E+00 | 0.030 | 0.015 | 4.09E-02 | -0.037 | 0.018 | 4.10E-02 | 1.08E-01 | 20 |
| **TARBP2** | **rs10876550** | **0.050** | **0.008** | **4.74E-11** | **-0.035** | **0.013** | **6.47E-03** | **-0.697** | **0.277** | **1.19E-02** | **1.68E-02** | **18** |
| TNFAIP8 | rs1035376 | 0.123 | 0.012 | 6.48E-25 | -0.048 | 0.018 | 6.92E-03 | -0.387 | 0.148 | 8.99E-03 | 8.62E-01 | 20 |

| **eTable 9. Coloc colocalization analysis** | | | | | | | |
| --- | --- | --- | --- | --- | --- | --- | --- |
| exposure | outcome | nsnps | PP.H0.abf | PP.H1.abf | PP.H2.abf | PP.H3.abf | PP.H4.abf |
| **CACYBP** | **atopic dermatitis** | **548** | **2.753E-41** | **0.050291856** | **1.7564E-41** | **0.0311644** | **0.918543787** |
| CALCOCO2 | atopic dermatitis | 732 | 1.824E-10 | 0.002195304 | 8.2791E-08 | 0.9965442 | 0.001260392 |
| CETN3 | atopic dermatitis | 570 | 2.486E-86 | 0.919173088 | 6.7513E-88 | 0.024907 | 0.055919946 |
| MOCS2 | atopic dermatitis | 1030 | 0 | 0.786085435 | 0 | 0.0347204 | 0.179194134 |
| PRRT3 | atopic dermatitis | 844 | 2.01E-126 | 0.700490513 | 3.989E-127 | 0.1387005 | 0.16080898 |
| PSTPIP2 | atopic dermatitis | 823 | 3.298E-81 | 0.886430209 | 1.6956E-82 | 0.0455047 | 0.068065052 |
| PVALB | atopic dermatitis | 980 | 0 | 8.5787E-06 | 0 | 0.9999909 | 4.99918E-07 |
| TARBP2 | atopic dermatitis | 432 | 2.269E-05 | 0.709601905 | 3.2841E-06 | 0.1025044 | 0.18786772 |
| TNFAIP8 | atopic dermatitis | 933 | 6.033E-19 | 0.681683389 | 8.0621E-20 | 0.0908671 | 0.227449549 |
| TNIP1 | atopic dermatitis | 1086 | 0.0050498 | 0.336968882 | 0.00782901 | 0.5222926 | 0.127859725 |


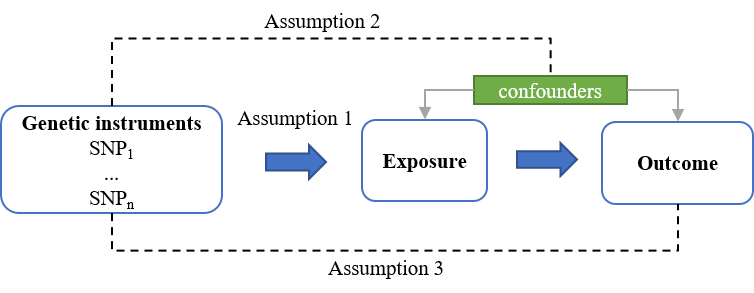


eFigure 1: The Theoretical Underpinnings and Principal Assumptions of Mendelian Randomization. This figure outlines the conceptual framework foundational to Mendelian Randomization (MR) studies, delineating its core assumptions critical for valid causal inference. Assumption 1 mandates that the genetic variant demonstrates a strong association with the exposure of interest. Assumption 2 requires that the instrumental genetic variants are not influenced by confounders that could affect the relationship between the exposure and outcome. Finally, Assumption 3 stipulates that the genetic variants impact the outcome solely through their effect on the exposure, ensuring a direct causal pathway.


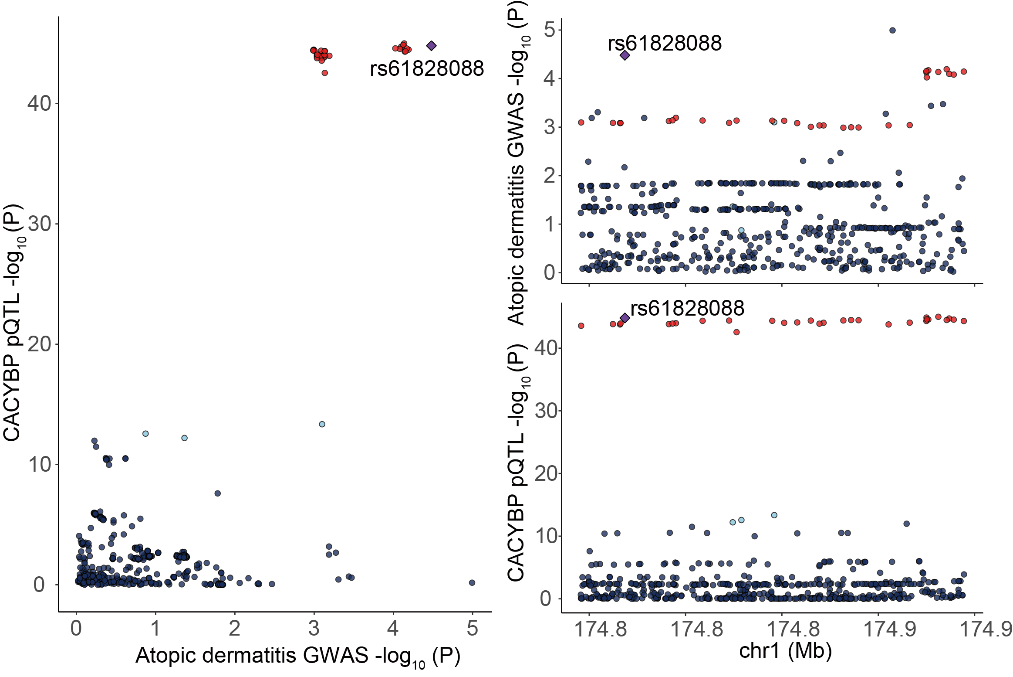


**eFigure** **2**: Bayesian Colocalization Analysis to Unveil Potential Causal Links Between Specific Plasma Proteins and Atopic Dermatitis. Analysis of CACYBP and Atopic Dermatitis: Focuses on the colocalization analysis for the CACYBP plasma protein with atopic dermatitis. Here, purple diamond-shaped markers similarly highlight SNPs with the smallest combined p-value from the respective protein GWAS and the GWAS of Atopic Dermatitis.


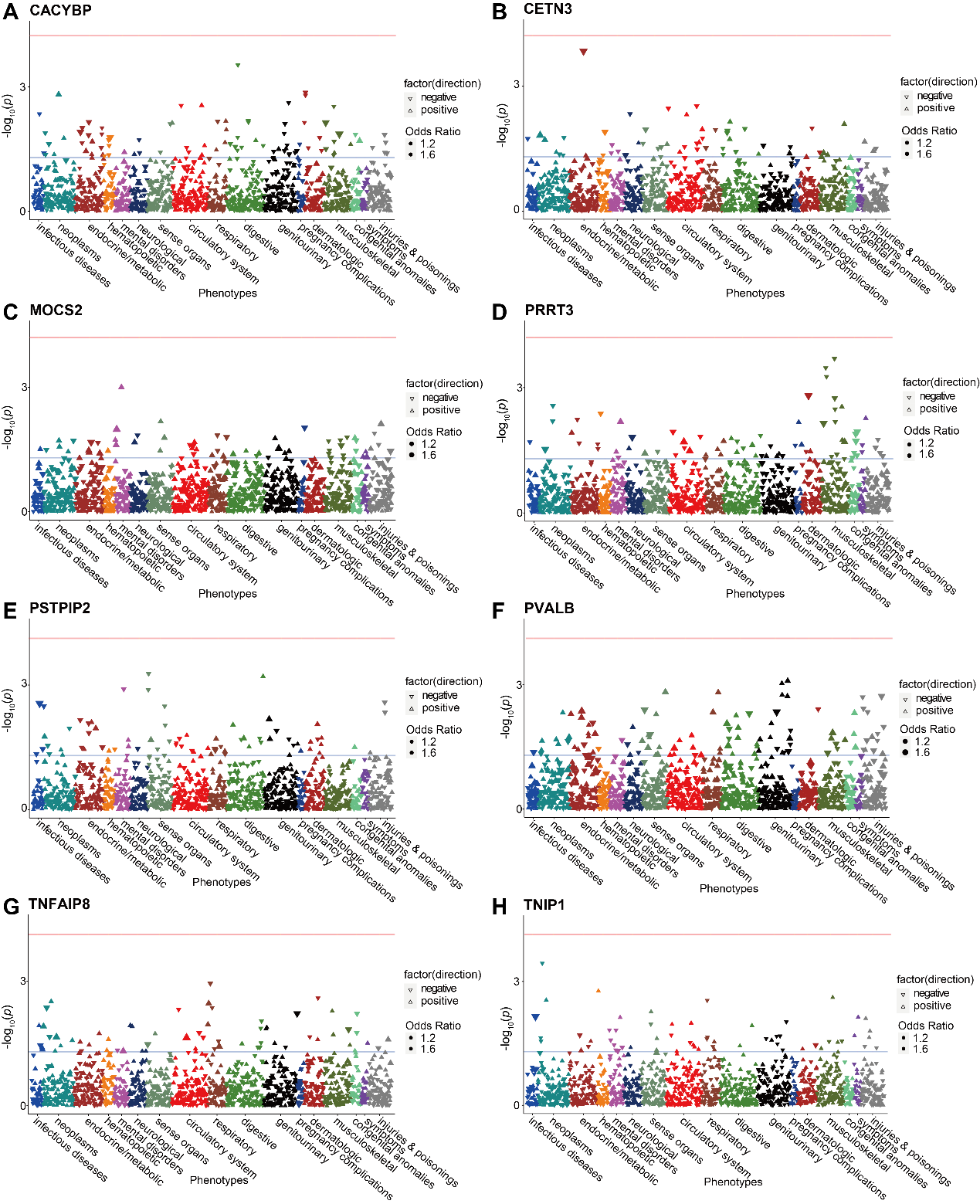


**eFigure 3**. Phenome-Wide Mendelian Randomization Analysis: Evaluating the Effects of Atopic Dermatitis-Associated Drug Targets Across Diverse Diseases. This Manhattan plot originates from a comprehensive phenome-wide Mendelian Randomization (MR) analysis, designed to assess the impacts of eight blood expression drug targets linked to atopic dermatitis (namely CACYBP, CETN3, MOCS2, PRRT3, PSTPIP2, PVALB, TNFAIP8, and TNIP1) on an extensive spectrum of 1,402 diseases documented in the UK Biobank (UKB). The y-axis meticulously catalogs the p-values generated from the phenome-wide MR study. A gray horizontal line demarcates the traditional p-value significance threshold (p < 0.05), while a pink line delineates an adjusted significance threshold (p < 3.56e-5), a correction for multiple comparisons across 1,402 tests. Each triangle marker on the plot represents an individual disease outcome, with the orientation of the triangle (upward for positive and downward for negative) visually encoding the directional causal influence exerted by the blood expression drug target on the disease in question.


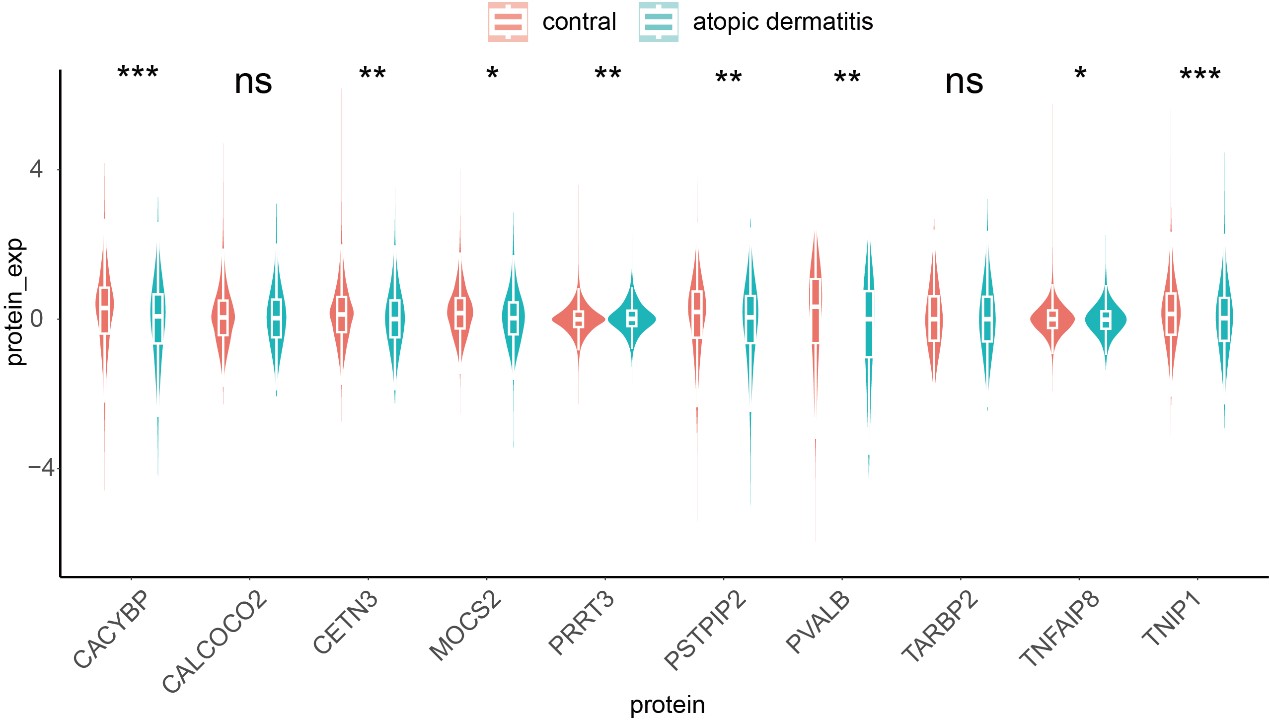


eFigure 4: Comparative Serum Protein Expression Analysis of Drug Targets in Atopic Dermatitis. This figure utilizes violin plots to depict the differential expression levels of serum proteins associated with five drug targets in inflammatory skin diseases. The color coding distinguishes between the normal control group (red) and patients atopic dermatitis Statistical significance is annotated as follows: ns denotes no significant difference; one asterisk (*) indicates p < 0.05; two asterisks (**) denote p < 0.01; three asterisks (***) represent p < 0.001; and four asterisks (****) signal p < 0.0001.


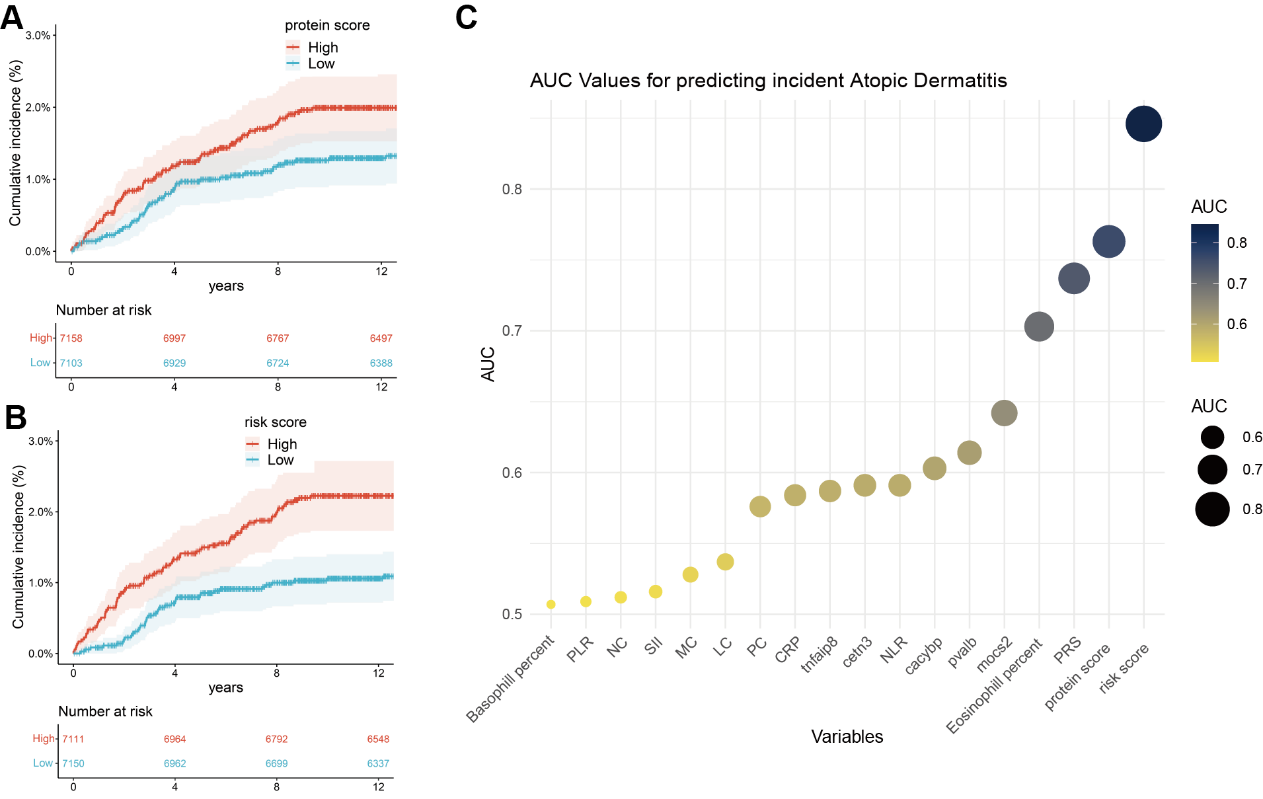


eFigure 5: Validation of Protein Scoring System for Atopic Dermatitis in the test set

A. Results of cumulative risk curve analysis of protein score in the test set.

B. Results of cumulative risk curve analysis of risk score in the test set.

C. AUC values of 5 protein markers, protein scores, multiple systemic inflammatory markers, and risk socre for predicting the onset of atopic dermatitis in the test set.
